# Blinatumomab as first salvage versus second or later salvage in adults with relapsed/refractory B‐cell precursor acute lymphoblastic leukemia: Results of a pooled analysis

**DOI:** 10.1002/cam4.3731

**Published:** 2021-03-18

**Authors:** Max S. Topp, Anthony S. Stein, Nicola Gökbuget, Heinz‐August Horst, Nicolas Boissel, Giovanni Martinelli, Hagop Kantarjian, Monika Brüggemann, Yuqi Chen, Gerhard Zugmaier

**Affiliations:** ^1^ Medizinische Klinik und Poliklinik II Universitätsklinikum Würzburg Würzburg Germany; ^2^ Gehr Leukemia Center City of Hope Medical Center Duarte CA USA; ^3^ Medizinische Klinik III (Hämatologie/Onkologie/Rheumatologie/Infektiologie Universitätsklinikum Frankfurt Germany; ^4^ Klinik für Innere Medizin II Universitätsklinikum Schleswig‐Holstein Kiel Germany; ^5^ Unité d'Hématologie Adolescents et Jeunes Adultes Hôpital Saint‐Louis Paris France; ^6^ Scientific Directorate Istituto Scientifico Romagnolo per lo Studio e la Cura dei Tumori (IRST) IRCCS Meldola (FC) Italy; ^7^ Department of Leukemia The University of Texas MD Anderson Cancer Center Houston Texas USA; ^8^ Sektion für Hämatologische Spezialdiagnostik Klinik für Innere Medizin II Universitätsklinikum Schleswig‐Holstein Kiel Germany; ^9^ Global Biostatistical Science Amgen Inc Thousand Oaks California USA; ^10^ Global Development Amgen Research (Munich) GmbH Munich Germany

**Keywords:** acute lymphoblastic leukemia, BiTE^®^, blinatumomab, salvage, stem cell transplant

## Abstract

**Background**

Blinatumomab is a BiTE^®^ immuno‐oncology therapy indicated for the treatment of patients with relapsed or refractory (r/r) B‐cell precursor (BCP) acute lymphoblastic leukemia (ALL).

**Aims:**

To assess the efficacy and safety of blinatumomab as first salvage versus second or later salvage in patients with r/r BCP ALL.

**Materials & Methods:**

Patient‐level pooled data were used for this analysis. In total, 532 adults with r/r BCP ALL treated with blinatumomab were included (first salvage, n = 165; second or later salvage, n = 367).

**Results:**

Compared with patients who received blinatumomab as second or later salvage, those who received blinatumomab as first salvage had a longer median overall survival (OS; 10.4 vs. 5.7 months; HR, 1.58; 95% CI, 1.26–1.97; *P* < .001) and relapse‐free survival (10.1 vs. 7.3 months; HR, 1.38; 95% CI, 0.98–1.93; *P* = .061), and higher rates of remission (n = 89 [54%] vs. n = 150 [41%]; odds ratio, 0.59; 95% CI, 0.41–0.85; *P* = .005), minimal residual disease response (n = 68 [41%] vs. n = 118 [32%]), and allogeneic hematopoietic stem cell transplant (alloHSCT) realization (n = 60 [36%] vs. n = 88 [24%]), and alloHSCT in continuous remission (n = 33 [20%] vs. n = 52 (14%]). In a subgroup analysis, there was no apparent effect of prior alloHSCT on median OS in either salvage group. The safety profile of blinatumomab was generally similar between the groups; however, cytokine release syndrome, febrile neutropenia, and infection were more frequent with second or later salvage than with first salvage.

**Discussion:**

In this pooled analysis, the logistic regression analyses indicated greater benefit with blinatumomab as first salvage than as second or later salvage, as evident by the longer median OS, longer median RFS, and higher rates of remission.

**Conclusion:**

Overall, blinatumomab was beneficial as first salvage and as second or later salvage, but the effects were favorable as first salvage.

## Graphical abstract

This pooled analysis of three clinical studies assessed the efficacy and safety of blinatumomab as first salvage versus second or later salvage in patients with relapsed or refractory (r/r) B‐cell precursor (BCP) acute lymphoblastic leukemia (ALL). Compared with patients who received blinatumomab as second or later salvage, those who received blinatumomab as first salvage had a longer median overall survival, relapse‐free survival, and higher rates of remission, minimal residual disease response, and allogeneic hematopoietic stem cell transplant (alloHSCT) realization. Overall, blinatumomab was beneficial as first salvage and as second or later salvage, but the effects were favorable as first salvage.

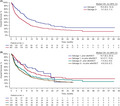

## 1 INTRODUCTION

Outcomes are poor among patients with relapsed or refractory (r/r) acute lymphoblastic leukemia (ALL). The reported complete remission (CR) rate among patients with r/r B‐cell precursor (BCP) ALL is 40% after first salvage, 21% after second salvage, and 11% after third or later salvage.^1^ One‐year survival rates among patients with r/r BCP ALL are 26% after first salvage, 14% after second salvage, and 12% after third or later salvage.^1^ Thus, there is an unmet need for effective salvage therapies in r/r BCP ALL.

Blinatumomab is a bispecific T‐cell engager (BiTE^®^ (bispecific T‐cell engager) immuno‐oncology therapy that directs cytotoxic T cells to lyse CD19‐positive B cells.^2^, ^3^, ^4^ In two open‐label, single‐arm, phase 2 studies of blinatumomab in patients with r/r BCP ALL, CR was achieved by 33%–42% of patients and the median overall survival (OS) was 6.1‐9.8 months.^5^, ^6^ In the randomized, open‐label, phase 3 TOWER study in patients (N = 405) with r/r Philadelphia chromosome–negative (Ph^−^) BCP ALL, salvage treatment with blinatumomab compared with chemotherapy was associated with longer OS (7.7 vs. 4.0 months; hazard ratio [HR], 0.71; 95% confidence interval [CI], 0.55‐0.93; *P* = .01) and a higher CR rate after 12 weeks of treatment (34% vs. 16%; *P* < .001).^7^ Given the ability of blinatumomab to bridge to allogeneic hematopoietic stem cell transplant (alloHSCT) in 24%‐67% of responders in these studies, there is potential for the improvement of OS among patients who achieve alloHSCT, particularly in later salvage.

This pooled analysis included the two phase 2 studies and the phase 3 TOWER study, and assessed the efficacy and safety of blinatumomab as first salvage or second or later salvage in patients with r/r BCP ALL.

## 2 MATERIALS AND METHODS

### 2.1 Patients, study design, and treatment

The patient eligibility criteria, study designs, and treatments in the three studies included in this pooled analysis were described previously.^5^, ^6^, ^7^ Patient‐level pooled data were used for this analysis. All three studies enrolled adults (aged ≥18 years) with Eastern Cooperative Oncology Group (ECOG) performance status ≤2. In addition, patients in the first phase 2 study (ClinicalTrials.gov, NCT01209286) had BCP ALL relapsed (reappearance of disease after CR lasting ≥28 days) after induction and consolidation or refractory (no CR) after induction and/or consolidation, >5% bone marrow blasts, and life expectancy ≥12 weeks; those with Ph^+^ ALL eligible for dasatinib or imatinib were excluded.^6^ Patients in the second phase 2 study (ClinicalTrials.gov, NCT01466179) had Ph^−^ BCP ALL primary refractory or relapsed (first relapse within 12 months of first remission, relapse within 12 months after alloHSCT, or no response to or relapse after first salvage therapy or beyond) and >10% bone marrow blasts.^5^ Patients in the phase 3 study (ClinicalTrials.gov, NCT02013167) had Ph^−^ BCP ALL refractory to primary induction therapy or to salvage with intensive combination chemotherapy, first relapse with the first remission lasting ≤12 months, second or greater relapse, or relapse at any time after alloHSCT and had >5% bone marrow blasts.^7^ Patients received blinatumomab in cycles of 4‐week continuous infusion followed by a 2‐week treatment‐free interval. Two induction cycles and up to three consolidation cycles were administered. Maintenance treatment was given every 12 weeks in the phase 3 study. Eligible patients received alloHSCT at the investigators’ discretion. Before each dose of blinatumomab, dexamethasone was given as prophylaxis for cytokine release syndrome (CRS) and neurologic events. All patients provided written, informed consent before enrollment. Institutional review board approval was obtained for each study.

### 2.2 Assessments

Response was assessed at the end of each treatment cycle. CR was defined as ≤5% bone marrow blasts and no evidence of disease was further defined by the extent of peripheral blood counts: CR with full hematologic recovery (platelets >100,000/μL and absolute neutrophil count (ANC) >1000/μL), CR with partial hematologic recovery (CRh; platelets >50,000/μL and ANC >500/μL), or CR with incomplete hematologic recovery (CRi; platelets >100,000/μL or ANC >500/μL). Blast‐free hypoplastic or aplastic bone marrow was defined as ≤5% bone marrow blasts, no evidence of disease, and insufficient recovery (platelets ≤50,000/μL and/or ANC ≤500/μL). Partial remission (PR) was defined as bone marrow blasts 6%‐25% with a ≥ 50% reduction from baseline. Progressive disease was defined as ≥25% increase from baseline in bone marrow blasts or absolute increase from baseline in circulating leukemic cells of ≥5000/μL. Relapse was defined as >5% bone marrow or peripheral blood blasts after CR/CRh/CRi. Minimal residual disease (MRD) response was defined as <10^−4^ detectable blasts per allele‐specific real‐time quantitative polymerase chain reaction.^8^, ^9^ All adverse events (AEs) were recorded and graded per the National Cancer Institute Common Terminology Criteria for Adverse Events, version 4.0.

### 2.3 Statistical analyses

Patient incidences of response rates were calculated and accompanied by two‐sided 95% exact binomial CIs. Time‐to‐event estimates were calculated using the Kaplan–Meier method. OS was defined as the time from first blinatumomab dose to death. Patients alive were censored at the last date known to be alive. Relapse‐free survival (RFS) was defined as the time from first CR or CRh within the first two cycles of treatment to hematologic or extramedullary relapse or death. Patients alive in remission were censored at the date of last assessment. OS was compared between patients who received blinatumomab as first salvage and those who received blinatumomab as second or later salvage using an unstratified Cox regression model. CR/CRh rates after two cycles of treatment were compared between patients who received blinatumomab as first salvage versus those who received blinatumomab as second or later salvage using an unstratified logistic regression model. *P*‐values <.05 were considered significant.

## 3 RESULTS

### 3.1 Patients

Overall, 532 patients from three clinical studies of blinatumomab in patients with r/r BCP ALL were included in this pooled analysis.^5^, ^6^, ^7^ Among these, 165 received blinatumomab as first salvage and 367 received blinatumomab as second or later salvage. Patients who received blinatumomab as first salvage tended to be older than those who received blinatumomab as second or later salvage (45 vs. 34 years) and had slightly better ECOG performance status (Table 1. Notable proportions of patients at study entry had prior alloHSCT (first salvage, 25%; second or later salvage, 38% and ≥50% bone marrow blasts (first salvage, 60%; second or later salvage, 68%.

**TABLE 1:** Demographics and baseline disease characteristics.

Characteristics	Salvage 1 (n = 165)	Salvage 2+ (n = 367)
Median (range) age, years	45 (19–80)	34 (18–77)
Sex, n (%)		
Men	98 (59)	225 (61)
Women	67 (41)	142 (39)
Race, n (%)		
White	144 (87)	290 (79)
Asian	7 (4)	21 (6)
Black	3 (2)	9 (3)
Other	6 (4)	18 (5)
Unknown	5 (3)	20 (5)
ECOG performance status, n (%)		
0	77 (47)	111 (30)
1	72 (44)	191 (52)
2	16 (10)	63 (17)
Unknown	0	2 (1)
Prior alloHSCT, n (%)	41 (25)	141 (38)
Median (range) bone marrow blasts at screening,^a^ %	78 (1–100)	81 (2–100)
Bone marrow blasts, n (%)		
≤5%	8 (5)	8 (2)
>5%–<10%	9 (6)	13 (4)
10%–<50%	35 (21)	78 (21)
≥50%	99 (60)	250 (68)
Unknown	14 (9)	18 (5)

Notes

Abbreviations: ECOG, Eastern Cooperative Oncology Group; alloHSCT, allogeneic hematopoietic stem cell transplant.

^a^ Based on central laboratory screening results.

### 3.2 Median OS and RFS

**FIGURE 1:**
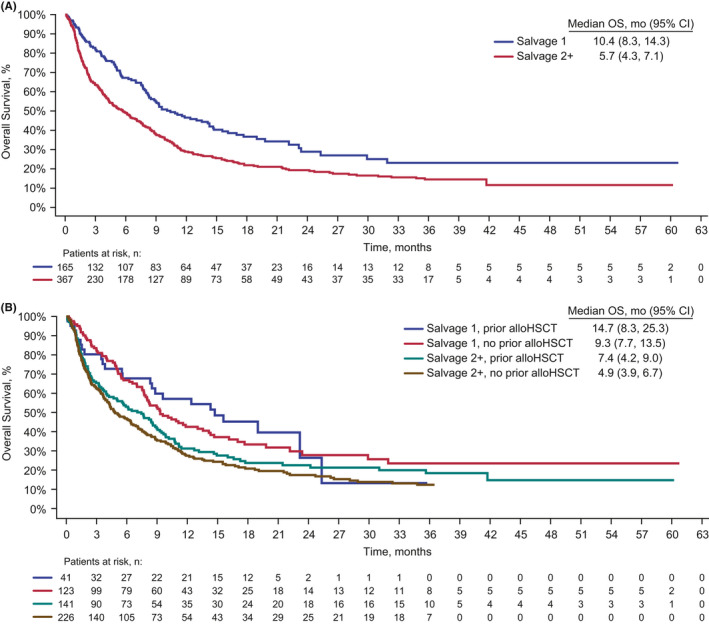
Kaplan–Meier estimated OS among patients who received blinatumomab as first salvage or second or later salvage in the overall population (A) or in subgroups by prior alloHSCT (yes vs. no; B). alloHSCT, allogeneic hematopoietic stem cell transplant; CI, confidence interval; OS, overall survival.

Patients who received blinatumomab as second or later salvage had significantly shorter OS than patients who received blinatumomab as first salvage (HR, 1.58; 95% CI, 1.26–1.97; *P* < .001; Figure 1A). The median OS was 5.7 months (95% CI, 4.3–7.1) among those who received blinatumomab as second or later salvage and 10.4 months (95% CI, 8.3–14.3) among those who received blinatumomab as first salvage. The estimated OS rates among patients who received blinatumomab as second or later salvage compared with first salvage were 29% and 47%, respectively, at 12 months, 19% and 29% at 24 months, and 12% and 23% at 60 months.

In a subgroup analysis, for patients who received blinatumomab as first salvage or as second or later salvage, median OS appeared to be shorter among those without prior alloHSCT (Figure 1B). Among patients who received blinatumomab as first salvage, median OS was 14.7 months (95% CI, 8.3–25.3) for those with prior alloHSCT and 9.3 months (95% CI, 7.7–13.5) for those without prior HSCT (Figure 1B). Among patients who received blinatumomab as second or later salvage, median OS was 7.4 months (95% CI, 4.2–9.0) for those with prior alloHSCT and 4.9 months (95% CI, 3.9–6.7) for those without prior alloHSCT (Figure 1B). The overlapping confidence intervals suggest that these data do not support a difference in OS based on prior alloHSCT status for either first or later salvage subgroups.

**FIGURE 2:**
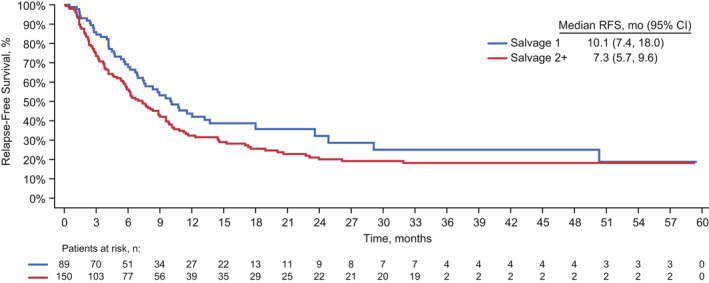
Kaplan–Meier estimated relapse‐free survival among patients who received blinatumomab as first salvage or second or later salvage. CI, confidence interval; OS, overall survival.

Among patients who achieved CR/CRh after two cycles of blinatumomab treatment (n = 239), 159 patients had disease relapse, disease progression, or had died (first salvage, n = 50; second or later salvage, n = 109); 80 were censored (first salvage, n = 39; second or later salvage, n = 41). There was no statistically significant difference between RFS among patients who received blinatumomab as first salvage compared with those who received blinatumomab as second or later salvage (HR, 1.38; 95% CI, 0.98–1.93; *p* = 0.061; Figure 2). The median RFS was 10.1 months (95% CI, 7.4–18.0) among patients who received blinatumomab as first salvage and 7.3 months (95% CI, 5.7–9.6) among those who received blinatumomab as second or later salvage. The estimated RFS rates among patients who received blinatumomab as first salvage compared with those who received blinatumomab as second or later salvage, respectively, were 44% and 32% at 12 months, 32% and 20% at 24 months, and 25% and 18% at 48 months.

### 3.3 Response and transplant realization

Patients who received blinatumomab as second or later salvage were less likely to achieve CR or CRh after two cycles than those who received blinatumomab as first salvage (odds ratio [OR], 0.59; 95% CI, 0.41–0.85; *p* = 0.005). CR or CRh after two cycles was achieved by 150 of 367 (41%; 95% CI, 36–46) patients who received blinatumomab as second or later salvage and by 89 of 165 (54%; 95% CI, 46–62) patients who received blinatumomab as first salvage (Table 2). CR was achieved by 101 (28%) patients who received blinatumomab as second or later salvage and 78 (47%) patients who received blinatumomab as first salvage. CRh was achieved by 49 (13%) patients who received blinatumomab as second or later salvage and 11 (7%) patients who received blinatumomab as first salvage. PR was achieved by nine (3%) patients who received blinatumomab as second or later salvage and four (2%) patients who received blinatumomab as first salvage.

**TABLE 2:** Best response, MRD response, and transplant realization.

	Salvage 1 (n = 165)			Salvage 2+ (n = 367)		
	n	%	95% CI	n	%	95% CI
Best response after two cycles						
CR or CRh	89	54	46–62	150	41	36–46
CR	78	47	40–55	101	28	23–32
CRh	11	7	3–12	49	13	10–17
CRi	1	1	0–3	3	1	<1–2
Blast‐free hypoplastic or aplastic bone marrow	6	4	1–8	24	7	4–10
Partial remission	4	2	1–6	9	3	1–5
Non‐response or unevaluable/missing post‐baseline assessment	46	28	21–35	136	37	32–42
Progressive disease	18	11	7–17	42	11	8–15
MRD response after two cycles^a^						
MRD response	68	41	34–49	118	32	27–37
MRD response among patients with CR/CRh	63	71	60–80	106	71	63
Patients with alloHSCT	60	36	29–44	88	24	20–29
Patients transplanted in continuous remission post‐blinatumomab	33	20	14–27	52	14	11–18
Patients with anti‐leukemic treatment other than blinatumomab	42	26	19–33	61	17	13–21
Patients transplanted after relapse post‐blinatumomab and/or refractory post‐blinatumomab	4	2	1–6	9	3	1–4

Notes

Abbreviations: alloHSCT, allogeneic hematopoietic stem cell transplant; CI, confidence interval; CR, complete remission with full hematologic recovery; CRh, CR with partial hematologic recovery; CRi, CR with incomplete hematologic recovery; MRD, minimal residual disease.

^a^ Bone marrow blasts <10^−4^.

Overall, MRD response was achieved by 68 (41%; 95% CI, 34–49) patients who received blinatumomab as first salvage and 118 (32%; 95% CI, 27–37) patients who received blinatumomab as second or later salvage (Table 2). Among those with CR or CRh, MRD response was achieved by 63 (71%; 95% CI, 60–80) patients who received blinatumomab as first salvage and 106 (71%; 95% CI, 63–78) patients who received blinatumomab as second or later salvage (Table 2). The rate of MRD response in patients with CR/CRh was not different between those who received blinatumomab as first salvage and those who received blinatumomab as second or later salvage.

**FIGURE 3:**
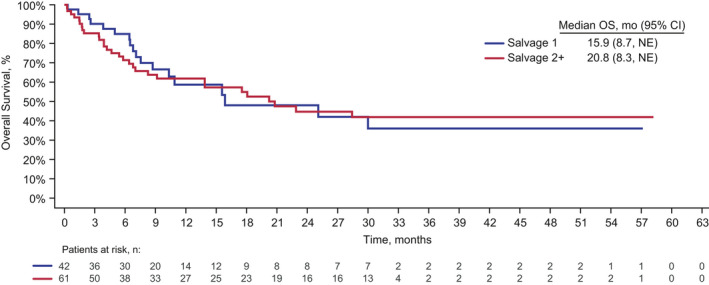
Kaplan–Meier estimated OS among patients who received blinatumomab as first salvage or second or later salvage followed by allogeneic hematopoietic stem cell transplant. CI, confidence interval; NE, not estimable; OS, overall survival.

Sixty (36%) patients who received blinatumomab as first salvage and 88 (24%) patients who received blinatumomab as second or later salvage went on to receive alloHSCT, including 42 (26%) and 61 (17%), respectively, who were in remission after two cycles (Table 2). Thirty‐three (20%) patients who received blinatumomab as first salvage and 52 (14%) patients who received blinatumomab as second or later salvage received alloHSCT during remission without additional anticancer therapy. There was no apparent difference in median OS among patients who received blinatumomab as first salvage or second and later salvage who went on to receive alloHSCT (Figure 3).

### 3.4 Adverse events

The incidence rate of treatment‐emergent AEs was consistent between patients who received blinatumomab as first salvage or as second or later salvage (99% vs. 99%; Table 3). The incidence rate of grade ≥3 treatment‐emergent AEs was also similar between patients who received blinatumomab as first salvage or as second or later salvage (81% vs. 85%). The incidences of the most commonly occurring (in ≥10% of patients) AEs of any grade and their respective grade ≥3 incidences are summarized in Table 3. The proportions of patients with grade ≥3 AEs of interest among those who received blinatumomab as first salvage or as second or later salvage were as follows: neurologic events (13% vs. 15%), CRS (28% vs. 38%), infection (28% vs. 38%), neutropenia (20% vs. 15%), and febrile neutropenia (18% vs. 24%).

**TABLE 3:** Summary of adverse events.

	Salvage 1 (n = 164)		Salvage 2+ (n = 364)	
	Any grade	Grade ≥3	Any grade	Grade ≥3
Patients with any treatment‐emergent AE, n (%)	162 (99)	133 (81)	361 (99)	310 (85)
Patients with any treatment‐emergent serious AE, n (%)	99 (60)		239 (66)	
Patients with a fatal treatment‐emergent AE, n (%)	16 (10)		76 (21)	
Patients with a fatal treatment‐related AE, n (%)	3 (2)^a^		10 (3)^b^	
AEs occurring in ≥10% of patients, n (%)				
Pyrexia	115 (70)	16 (10)	209 (57)	26 (7)
Headache	55 (34)	3 (2)	119 (33)	7 (2)
Anemia	42 (26)	32 (20)	73 (20)	54 (15)
Nausea	37 (23)	0	78 (21)	0
Neutropenia	36 (22)	33 (20)	60 (17)	54 (15)
Fatigue	35 (21)	1 (1)	53 (15)	7 (2)
Febrile neutropenia	33 (20)	29 (18)	97 (27)	88 (24)
Diarrhea	32 (20)	1 (1)	81 (22)	5 (1)
Peripheral edema	31 (19)	2 (1)	77 (21)	3 (1)
Constipation	30 (18)	0	55 (15)	1 (<1)
Thrombocytopenia	30 (18)	25 (15)	52 (14)	41 (11)
Cough	29 (18)	0	59 (16)	1 (<1)
Vomiting	29 (18)	0	42 (12)	0
Hypokalemia	26 (16)	5 (3)	80 (22)	23 (6)
Rash	22 (13)	0	32 (9)	3 (1)
Tremor	22 (13)	3 (2)	53 (15)	2 (1)
Insomnia	21 (13)	0	43 (12)	1 (<1)
Back pain	20 (12)	1 (1)	51 (14)	9 (3)
Bone pain	18 (11)	2 (1)	38 (10)	13 (4)
Pain in extremity	18 (11)	3 (2)	37 (10)	3 (1)
Cytokine release syndrome	17 (10)	4 (2)	51 (14)	9 (3)
Asthenia	16 (10)	3 (2)	27 (7)	6 (2)
Chills	16 (10)	0	45 (12)	1 (<1)
Hyperglycemia	15 (9)	3 (2)	37 (10)	22 (6)
Dizziness	14 (9)	0	38 (10)	2 (1)
Hypomagnesemia	12 (7)	0	45 (12)	1 (<1)
Alanine aminotransferase increased	11 (7)	5 (3)	45 (12)	26 (7)
Abdominal pain	8 (5)	2 (1)	49 (14)	10 (3)
Aspartate aminotransferase increased	7 (4)	2 (1)	40 (11)	18 (5)

Notes

Abbreviation: AE, adverse event.

^a^ Bronchopulmonary aspergillosis (n = 1), central nervous system infection (n = 1), and sepsis syndrome (n = 1).

^b^ Sepsis (n = 3), acute respiratory failure, bacterial infection, *Candida* infection, encephalopathy, *Escherichia* sepsis, neutropenic sepsis, and respiratory failure (n = 1 each).

The incidence rate of serious treatment‐emergent AEs was somewhat lower among patients who received blinatumomab as first salvage compared with those who received blinatumomab as second or later salvage (60% vs. 66%), as was the frequency of discontinuations due to AEs (11% vs. 20%). The proportion of patients with fatal treatment‐emergent AEs was lower among those who received blinatumomab as first salvage compared with second or later salvage (10% vs. 21%; Table 3); however, the proportion of patients with treatment‐related fatal AEs was similar (2% vs. 3%). The three treatment‐related fatal AEs occurring among patients who received blinatumomab as first salvage were bronchopulmonary aspergillosis, central nervous system infection, and sepsis syndrome. The 10 treatment‐related fatal AEs occurring among patients who received blinatumomab as second or later salvage were sepsis, acute respiratory failure, bacterial infection, *Candida* infection, encephalopathy, *Escherichia* sepsis, neutropenic sepsis, and respiratory failure.

## 4 DISCUSSION

The randomized, open‐label phase 3 TOWER study demonstrated significantly longer median OS and higher rates of CR with blinatumomab versus chemotherapy in patients with r/r Ph^−^ BCP ALL.^7^ Two prior phase 2 studies also showed efficacy with single‐agent blinatumomab in patients with r/r BCP ALL.^5^, ^6^ In this pooled analysis (N = 532) of the two phase 2 studies and the TOWER study, blinatumomab was effective as first salvage and as second or later salvage. Notably, the logistic regression analyses indicated greater benefit with blinatumomab as first salvage than as second or later salvage, as evident by the longer median OS (10.4 vs. 5.7 months; HR, 1.58; *p* < 0.001), longer median RFS (10.1 vs. 7.3 months; HR, 1.38; *p* = 0.061), and higher rates of remission (54% vs. 41%; OR, 0.59; *p* = 0.005). Other studies have also shown better outcomes in patients who received blinatumomab a first salvage compared with those who received blinatumomab as second or later salvage.^1^, ^10^, ^11^

Disease and patient characteristics have a considerable impact on response to treatment and outcome in patients with r/r BCP ALL. A large proportion (92%) of patients included in this pooled analysis was required to be either refractory or to have disease relapse within 1 year of first remission. In multivariate analyses, poor disease status at the time of salvage (e.g., refractory with prior transplant) and relapse within the first year of CR have been associated with shorter OS.^1^, ^12^ Notable proportions of patients in this analysis had received prior alloHSCT (first salvage, 25%; second or later salvage, 38%) or had ≥50% bone marrow blasts (first salvage, 60%; second or later salvage, 68%). Prior alloHSCT and higher levels of bone marrow blasts or white blood cells have each been associated with a shorter OS in patients with r/r BCP ALL.^1^, ^12^, ^13^, ^14^ However, in the subgroup analysis presented here, there was no apparent effect of prior alloHSCT on median OS among patients who received blinatumomab as first salvage or as second or later salvage.

MRD response is a predictor of outcomes in BCP ALL.^15^, ^16^ Achievement of MRD response with first salvage, but not with second salvage, has been associated with a longer OS and event‐free survival in patients with r/r BCP ALL.^16^ In this analysis, an MRD response occurred in 71% of patients with CR or CRh who received blinatumomab as first salvage or as second or later salvage, indicating further the potential for blinatumomab efficacy in later salvage in patients with r/r BCP ALL.

Inducing a remission followed by HSCT is the primary goal of salvage therapy in patients with r/r Ph^−^ BCP ALL.^17^ In this analysis, 36% of patients who received blinatumomab as first salvage and 24% of patients who received blinatumomab as second or later salvage subsequently received alloHSCT, including 20% and 14%, respectively, who were in continuous remission. In comparison, a retrospective analysis of study groups and centers in Europe and the United States found that 28% of patients with r/r Ph^−^ BCP ALL received HSCT after first salvage, 49% of whom were in CR at the time of transplant.^1^ The alloHSCT realization rates in this analysis are encouraging, particularly given the advanced disease of this patient population, and indicate that blinatumomab is effective at bridging to transplant both as first salvage and as second or later salvage.

The safety profile of blinatumomab was generally similar among patients treated as first salvage and those treated with blinatumomab as second or later salvage in this pooled analysis. However, the incidence rate of serious treatment‐emergent AEs was slightly higher among patients treated in second or later salvage compared with first salvage (66% vs. 60%), as was the frequency of treatment‐emergent fatal AEs (21% vs. 10%). Notably, however, there was no appreciable difference between the groups in the proportion of treatment‐related fatal AEs (first salvage, 2%; second or later salvage, 3%). Certain AEs of interest were more common among patients who received blinatumomab as second or later salvage compared with those who received blinatumomab as first salvage (CRS, infection, and febrile neutropenia), whereas others were not (neurologic events and neutropenia). These differences are not surprising given that patients receiving later salvage often have more advanced disease, poorer prognosis, and poorer performance status than patients receiving earlier lines of therapy. The occurrence of neurologic events and CRS does not preclude treatment with blinatumomab since these were managed successfully with dexamethasone and treatment interruption in the studies included in this analysis ^5^, ^6^, ^7^ and in other studies of blinatumomab.^18^, ^19^, ^20^

There are a few limitations of this pooled analysis that should be considered. First, because of the design of the studies included, there was an imbalance in the number of patients who received blinatumomab as first salvage (n = 165) compared with those who received blinatumomab as second or later salvage therapy (n = 367). Second, patients with Ph^+^ ALL were not excluded from enrollment in the first phase 2 study^6^; however, only two patients overall in the analysis had Ph^+^ BCP ALL. Finally, the impact of prior inotuzumab ozogamicin (INO), an anti‐CD22 monoclonal antibody–calicheamicin conjugate, was not evaluated in this study as INO was approved for the treatment of adults with r/r BCP ALL after the studies reported here.^21^ Clinical trials evaluating the sequencing and combination of blinatumomab with INO are ongoing NIH––National Cancer Institute^22^: https://www.cancer.gov/about‐cancer/treatment/clinical‐trials/intervention/inotuzumab‐ozogamicin).

In conclusion, although blinatumomab as first salvage and as second or later salvage induced remission, bridged to HSCT, and showed benefits in median OS and RFS in this population of patients with r/r BCP ALL, the greatest benefit was for blinatumomab as first salvage.

## 5 DATA SHARING AND ACCESSIBILITY

Qualified researchers may request data from Amgen clinical trials. Complete details are available at http://www.amgen.com/datasharing.

## CONFLICT OF INTEREST

Max S. Topp: received fees for serving on the advisory boards of Amgen Inc., Regeneron, Affimed, Jazz Pharmaceuticals, Gilead Sciences, and Pfizer, and travel support from Amgen Inc., Roche, Regeneron, and Affimed.

Anthony S. Stein: participated in speakers’ bureau for Amgen Inc., Celgene, and Stemline.

Nicola Gökbuget: served on the advisory board and speakers’ bureau of, and received research funding from Amgen Inc.; served on the advisory board and speakers’ bureau of, and received travel support from Pfizer.

Heinz‐August Horst: received research funding, travel support from, and participated in advisory boards for Amgen Inc.; participated in advisory board for Pfizer, Jazz Pharmaceuticals, and Novartis; and received research funding from Regeneron.

Nicolas Boissel: provides consultancy to Amgen Inc.

Giovanni Martinelli: no disclosures.

Hagop Kantarjian: received research funding from AbbVie, Agios, Amgen Inc., Ariad, Astex, Bristol‐Myers Squibb, Cyclacel, Daiichi‐Sankyo, ImmunoGen, Jazz Pharmaceuticals, Novartis, and Pfizer; and received honoraria from AbbVie, Actinium, Agios, Amgen Inc., Pfizer, and Takeda.

Monika Brüggemann: received consulting fees and honoraria from Amgen Inc., Celgene, Incyte, Janssen, and Roche, and research funding from Affimed, Regeneron, and Roche.

Yuqi Chen is an employee of and owns stock in Amgen Inc.

Gerhard Zugmaier is an employee of, has patents from, and owns stock in Amgen Inc.

## AUTHOR CONTRIBUTIONS

Max S. Topp: recruited patients, interpreted data, and revised or wrote the manuscript.

Anthony S. Stein: recruited patients, performed research, interpreted data, and revised or wrote the manuscript.

Nicola Gökbuget: recruited patients, interpreted data, and revised or wrote the manuscript.

Heinz‐August Horst: recruited patients, performed central morphology, and revised or wrote the manuscript.

Nicolas Boissel: recuited patients and revised or wrote the manuscript.

Giovanni Martinelli: recruited patients and revised or wrote the manuscript.

Hagop Kantarjian: recruited patients, interpreted data, and revised or wrote the manuscript.

Monika Brüggemann: performed the research and central MRD analyses, and revised or wrote the manuscript.

Yuqi Chen: analyzed data, interpreted data, and revised or wrote the manuscript.

Gerhard Zugmaier: designed the trial, performed research, analyzed data, and revised or wrote the manuscript.
